# Monitoring Approach of Fatality Risk Factors for Patients with Severe Acute Pancreatitis Admitted to the Intensive Care Unit. A Retrospective, Monocentric Study

**DOI:** 10.3390/diagnostics11112013

**Published:** 2021-10-29

**Authors:** Tudorel Mihoc, Cristi Tarta, Ciprian Duta, Raluca Lupusoru, Greta Dancu, Monica Anca Oprescu-Macovei, Amadeus Dobrescu, Fulger Lazar

**Affiliations:** 1 Department X, Surgical Emergencies Clinic, “Victor Babeș” University of Medicine and Pharmacy Timișoara, Eftimie Murgu Sq. No. 2, 300041 Timișoara, Romania; tudorel_mihoc@yahoo.com; 2 Department X, 2nd Surgical Clinic, Researching Future Chirurgie 2, “Victor Babeș” University of Medicine and Pharmacy Timișoara, Eftimie Murgu Sq. No. 2, 300041 Timișoara, Romania; ciprian_duta@yahoo.com (C.D.); amadeusdobrescu@yahoo.com (A.D.); lazarfulger@yahoo.com (F.L.); 3 Department VII, Gastroenterology, “Victor Babeș” University of Medicine and Pharmacy Timișoara, Eftimie Murgu Sq. No. 2, 300041 Timișoara, Romania; raluca_lupusoru@yahoo.ro (R.L.); gretadancu@yahoo.com (G.D.); 4 Department of Functional Sciences, Centre for Modeling Biological Systems and Data Analysis, “Victor Babes” University of Medicine and Pharmacy, 300041 Timișoara, Romania; 5 Gastroenterology, Emergency Hospital Prof. Dr. Agripa Ionescu, UMF Carol Davila, 011356 București, Romania

**Keywords:** severe acute pancreatitis, intensive care unit, neutrophils/lymphocytes ratio, platelets/lymphocytes ratio

## Abstract

Acute pancreatitis is an unpredictable disease affecting the pancreas and it is characterized by a wide range of symptoms and modified lab tests, thus there is a continuing struggle to classify this disease and to find risk factors associated with a worse outcome. The main objective of this study was to identify the risk factors associated with the fatal outcome of the intensive care unit’s patients diagnosed and admitted for severe acute pancreatitis, the secondary objective was to investigate the prediction value for the death of different inflammatory markers at the time of their admission to the hospital. This retrospective study included all the patients with a diagnosis of acute pancreatitis admitted to the Intensive Care Unit of the Emergency County Hospital Timisoara between 1 January 2016 and 31 May 2021. The study included 53 patients diagnosed with severe acute pancreatitis, out of which 21 (39.6%) survived and 32 (60.4%) died. For the neutrophils/lymphocytes ratio, a cut-off value of 12.4 was found. When analyzing age, we found out that age above 52 years old can predict mortality, and for the platelets/lymphocytes ratio, a cut-off value of 127 was found. Combining the three factors we get a new model for predicting mortality, with an increased performance, AUROC = 0.95, *p* < 0.001. Multiple persistent organ failure, age over 50, higher values of C reactive protein, and surgery were risk factors for death in the patients with severe acute pancreatitis admitted to the intensive care unit. The model design from the neutrophils/lymphocytes ratio, platelets/lymphocytes ratio, and age proved to be the best in predicting mortality in severe acute pancreatitis.

## 1. Introduction

Acute pancreatitis (AP) is an unpredictable disease affecting the pancreas and it is characterized by a wide range of symptoms and modified lab tests, thus there is a continuous struggle to classify this disease and to find the risk factors associated with a worse outcome in order to act early and improve outcome.

AP is a great burden for healthcare systems around the globe, being the leading cause of aggregates costs in United States, second in hospitalization days, and the fifth cause for in-hospital deaths [1]. In accordance with the Revised Atlanta Classification for AP, the majority of patients have a mild form of the disease [2], but up to 25% will develop severe acute pancreatitis (SAP) with a mortality rate that could rise over 50% [3].

Studies have suggested that an additional severity grade should be added to the ones which are already in use; they called this extra severity grade *Critical Acute Pancreatitis*. This new severity grade includes those cases of AP with a much higher mortality rate due to the association of persistent organ failure and the infected (peri)pancreatic necrosis [4]. Other authors proposed that the AP with a severe setting on admission should be considered as a distinct entity and name it early severe acute pancreatitis, in which patients had at least one organ failure or sepsis or systemic inflammatory response syndrome (SIRS) at the time of admission to hospital [5].

It is of utmost importance to try to predict as early as possible the evolution of the disease, having in view that those patients who have a higher risk of developing a severe form of acute pancreatitis might benefit from an early transfer to the Intensive Care Unit (ICU) with an improved outcome. This led to the development of a multitude of score systems, but all of them reached a plateau of maximum effectiveness and some have become very complex but not easy to implement in daily practice at the hospital (Ranson, Osborne, Blamey, Imrie, Balthazar, Bedside Index for Severity in Acute Pancreatitis—BISAP, Acute Physiology and Chronic Health Evaluation II—APACHE II, Pancreatitis Outcome Prediction—POP, Sequential Organ Failure Assessment—SOFA, New Simplified Acute Physiology Score—SAPS II, and others).

Because the main mechanism of inflicting systemic damage in SAP is considered the SIRS, other predicting factors were searched due to their link with inflammation and ease availability: C reactive protein (CRP), white blood cells (WBC) elevation, lymphocytopenia, neutrophils-lymphocytes ratio (NLR), and platelets-lymphocytes ratio (PLR).

Thus, several studies showed that NLR and PLR were associated with the severity of acute pancreatitis at the time of their admission to hospital [6,7] and an elevated NLR was also linked with increased mortality in SAP [8,9]. CRP was considered an independent prognostic factor for morbidity and mortality in AP [6,9]. The majority of the studies involving AP were conducted on a heterogeneous population of patients regarding the types of AP, drawing conclusions usually for patients with SAP from a pool that mostly included moderate and mild forms of AP—which could be difficult and misleading. One reason was that the number of SAP cases is scarce in a regular hospital and scattered over long periods of time, in other words, making it, thus, difficult to have an adequate number of patients enrolled.

The main objective of this study was to find the risk factors associated with the fatal outcome of the intensive care unit’s patients diagnosed and admitted with SAP, the secondary objective was to investigate the prediction value for the death of different inflammatory markers at the time of their admission to the hospital.

## 2. Materials and Methods

### 2.1. Patients’ Population

This retrospective study included all the patients with a diagnosis of acute pancreatitis admitted to the Intensive Care Unit of County Emergency Hospital Pius Brinzeu Timisoara between 1 January 2016 and 31 December 2020. The data were extracted from a computer database and from patients’ files with the local ethics committee approval. First, the diagnosis of AP was searched according to International Classification of Disease 10 for acute pancreatitis—K85 and variants, adding the filter for admission to the ICU.

The revised Atlanta classification was used to determine the severity of AP [2]. The main causes of pancreatitis were considered as biliary (when confirmed by imaging), alcohol consumption, hypertriglyceridemia, or unknown (when no cause was found).

Inclusion criteria: patients with severe acute pancreatitis admitted to intensive care unit.

Exclusion criteria: patients with acute severe pancreatitis transferred from another hospital, the patients with AP as a secondary diagnosis, and the patients with milder forms than severe pancreatitis—according to the Atlanta Revised Classification.

Follow-up was 90 days after discharge and had as a primary outcome the survival of the patients.

### 2.2. Data Management

Organ failure was assessed according to the Marshal criteria and was considered persistent if it lasted for more than 48 h. Those patients who had AP with organ failures but died in less than 48 h from debut or admission, were considered to have had a persistent organ failure. If a patient had persistent organ failure concomitant with a transient organ failure, the patient was included in the single persistent organ failure group.

The CT scan severity index (CTSI) was calculated according to the Balthazar score [10] and when the result assessment was not previously performed (the index was not calculated) or was uncertain, the images were reviewed with a consultant radiologist using the image data from the hospital records.

The following patients’ demographics and clinical variables were extracted and recorded: gender, age, etiology, organ failure, CTSI, hospital stay, days in the ICU, surgical procedures, lipase, neutrophils-lymphocytes ratio at admission to the hospital and at admission to the ICU, platelets-lymphocytes ratio, CRP, death.

Due to the fact that different laboratory assessment equipment was used during this period of time, and some of them had different factory established normal values intervals for CRP and lipase, the ratio between the measured value and the maximum normal value of that assessing equipment was used in the study.

The first two weeks of AP are characterized by a systemic inflammatory response syndrome with secondary multi-organ failure owing to the release of various cytokines being the cause of death; after two weeks, the infection of pancreatic and peripancreatic necrosis and secondary multi-organ failure become the main cause of death, therefore, we decided to split the patients who died in our study in early deaths and late deaths based on this assumption.

### 2.3. Statistical Analysis

The statistical analysis was performed using MedCalc Version 19.3.1. Descriptive statistics were used for clinical, anthropometric, and demographic data of the patients. Numerical variables with normal distribution are presented as the means ± standard deviation, while variables with non-normal distribution are presented as median values and range. The Kolmogorov–Smirnov test was used for measuring the distribution of numerical variables. Qualitative variables were presented as numbers and percentages. Parametric tests (*t*-test) were used for the assessment of differences between numerical variables with normal distribution and nonparametric tests (Mann–Whitney or Kruskal–Wallis tests) for variables with non-normal distribution. Chi-square (X2) test (with Yates’ correction for continuity) was used for comparing proportions expressed as percentages (“n” designates the total number of subjects from a particular group). Confidence intervals (95%) were calculated for each predictive test and a *p*-value < 0.05 was considered as significant for each statistical test. The Pearson Test was used for correlations. Areas under the receiver-operating characteristic (AUROC) curves were calculated for the mortality predicting scores. In order to see if what we performed had enough power, a post hoc power analysis was conducted. A sample size of 53 subjects was used for the statistical analyses. The alpha level for this analysis was <0.05. The post hoc analysis revealed the statistical power for this study 0.80 for detecting a small effect size.

## 3. Results

**Figure 1:**
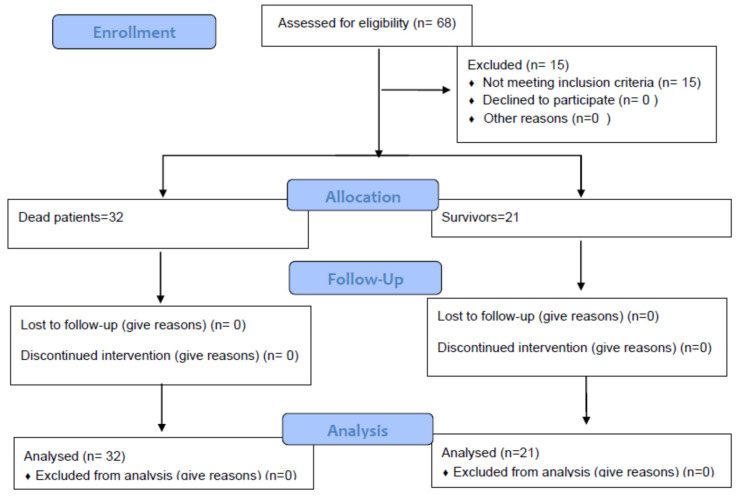
Study population flow chart.

During the study period, there were allocated 68 patients admitted to the ICU with AP. Fifteen patients were excluded from the study, as follows: eight patients because they had an acute moderate-severe form, three patients were transferred from other hospitals and no data were available for the early stages of the disease and, four had other diseases and AP was secondary illness (Figure 1).

Thus, the study included 53 ICU patients diagnosed with SAP, out of which 21 (39.6%) survived and 32 (60.4%) died, *p* = 0.14.

The patient’s characteristics are presented in Table 1.

According to the moment of death, two groups could be determined and compared. They were considered as early deaths if there were 14 days or less since admission to the hospital and late deaths if there were more than 14 days, data in Table 2.

In univariate analysis, the factors associated with the poor outcome were: NLR, PLR, surgery, age, and biliary etiology. NLR had an OR of 1.5 (0.78–2.2) with a *p*-value of 0.03, PLR with an OR of 1.0 (0.99–1.0) with a *p*-value of 0.01, surgery with an OR of 5.2 (0.99–35.14) with a *p*-value of 0.001, biliary etiology with an OR of 1.3 (0.75–1.92) with a *p*-value of 0.01, and age OR 1.1 (0.98–1.20) with a *p*-value of < 0.001.

**Figure 2:**
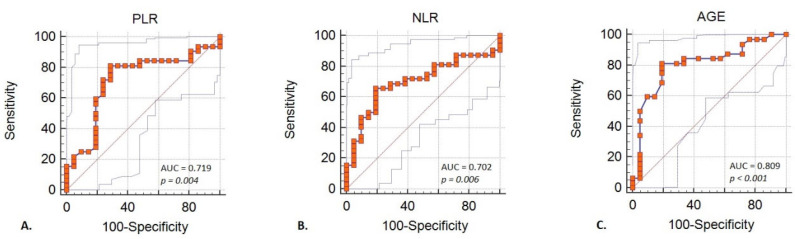
(**A**) Performance of PLR for predicting severe acute pancreatitis mortality according to ROC analysis; (**B**) performance of NLR for predicting severe acute pancreatitis mortality according to ROC analysis; (**C**) performance of AGE for predicting severe acute pancreatitis mortality according to ROC analysis. NLR—neutrophils-lymphocytes ratio; PLR—platelets-lymphocytes ratio; *p*—*p*-value.

**Figure 3:**
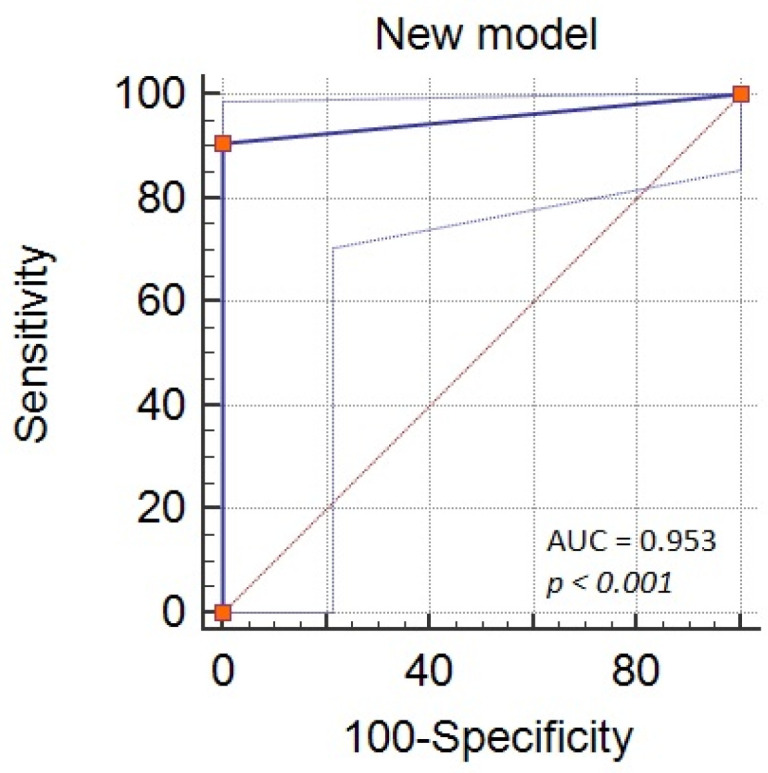
Performance of the combination (NLR >12.4 + PLR >127 + AGE >52) for predicting severe acute pancreatitis mortality according to ROC analysis. NLR—neutrophils-lymphocytes ratio; PLR—platelets-lymphocytes ratio; *p*—*p-*value.

With ROC analysis we aimed to identify the best cut-off value that can predict the mortality in severe acute pancreatitis. For NLR, a cut-off value of 12.4 was found. In regards to age, we found out that age above 52 can predict mortality. In addition, for PLR, a cut-off value of 127 was found (Table 3 and Figure 2). Combining the three factors we obtain a new model for predicting mortality, with an increased performance, AUROC = 0.95, *p* < 0.001, Se = 90.6%, Sp = 100% (Figure 3).

There were five patients operated on who survived during the study period; one had a laparoscopic cholecystectomy and external biliary drainage, thoracocentesis day seven after admission, another one had a simple cholecystectomy, the others three had two or three consecutive interventions addressing several collections. The other 20 patients who died and had a surgical procedure had eight abdominal decompressions for abdominal compartment syndrome one or two days after admission, the others had drainage of the infected pancreatic and peri-pancreatic necrosis performed between day 9 and day 78 after admission, among these patients eight had multiple reinterventions for peritonitis, bleeding in the peritoneal cavity, abscess recurrence, sigmoid necrosis, splenic infarction, and extensive infection of the soft tissues of the abdominal wall.

## 4. Discussion

Until the revised Atlanta classification in 2012, AP was mild and severe. After the revised Atlanta classification, the severity was set for three classes. There are some data in the literature that ask for another classification named determinant-based classification of acute pancreatitis severity for a better separation of severe cases of AP [4]. Three separated groups emerged from analyzing the patients with SAP: patients at higher risk of mortality due to persistent organ failure—early deaths, those without organ failure who are at higher risk of morbidity and mortality due to necrotizing pancreatitis and infection—late deaths, and those with prohibitive mortality when infected pancreatic necrosis and persistent organ failure both are present, for the later they proposed a new class called critical severe [4]. The hereby classification did not explain the rate of early deaths in our study—most of the patients died early and did not have the time to develop infected necrosis, based on our early death rate most critical forms of SAP were those determined by the SIRS and persistent early organ failure.

The high mortality rate for the patient admitted to the ICU in our study (60.4%) could be linked with a high percentage of critical patients at the in-hospital admission or soon after. Our study indicated a hospital stay of only 5 days for the patients who died; moreover, there were studies which have a mortality rate of 42% for the cases labeled as “early severe acute pancreatitis” which died early after admission [5]. The timing for developing severe acute pancreatitis with a fatal outcome is different for local factors (pancreatic necrosis with infection) when compared with the systemic factors (SIRS followed by transient or persistent organ failure) [11].

The systemic pathogenic factors determine the severity of the early phase of acute pancreatitis, characterized by the presence and persistence of organ failures due to the persistent SIRS caused by released inflammatory cytokines, and we still do not have the proper means or knowledge of how to efficiently fight back. Contrarily, there are the local factors that are predominantly found later on in pancreatitis’ evolution allowing us some time to take invasive procedural measures with the purpose of facilitating the healing process. Placing all these patients in one basket will produce a mixed population with different needs at a different course of the disease, making conclusions hazardous.

From our study, we found that “early-death” patients were more likely to die without a known etiology of the SAP (*p* = 0.01), had multiple organ failure (*p* = 0.04), and less surgical procedures (*p* = 0.001) when compared with the “late-death” patients.

The “late-death” group had a prolonged hospital staying before being admitted to the ICU compared with the survival group (22days versus 1 day), thus an early assessment and a different classification of severity of acute pancreatitis might have improved the survival chance by recognizing these patients as a different entity. At the same time, all the patients within the “late-death” group had at least one surgical procedure.

Surgery was one significant risk factor for death in our study, but surgical procedures cannot be evaluated with the current system of classification for AP, because in the “early-death” group, the procedures were usually performed on an acute abdomen with abdominal hypertension and patients with low responsiveness to resuscitation therapy, while in the “late-death” group, the surgery was addressing the local infectious complication.

More deaths were recorded in the first two weeks after admission to the hospital than after [12], a classic belief that in SAP the mortality occurrence graphic has two spikes, a higher early one in which the persistent organ failure is due to SIRS, and a second one when the organ failure is due to sepsis [13], but some stated that mortality in SAP is a continuing process with decreasing numbers from admission to the third week of disease [14]. Scheppers et al. found that there was no association between the duration or the onset of organ failure, the infected necrosis, and death rate in patients with SAP, but they were not able to find a cause for the early death either [14].

The most likely cause of the higher mortality rate—in our study, (60.4%)—was because of the lack of available beds in the ICU, therefore, only the severe acute pancreatitis with persistent organ failure and the need for mechanical ventilation were admitted to the ICU. Another cause could be that our hospital is a tertiary hospital for an area with more than 2 million people, all the severe cases from this area were referred to us, and the referral hospitals had a higher death rate [15].

Factors associated with a fatal outcome in our study were older age, higher CRP at hospital admission, unknown or biliary etiology, and surgery.

Age above 50 was a significant risk factor for death (*p* ≤ 0.0001) [16,17], this could be the result of an increased number of comorbidities in older people [18] or the greater susceptibility of the former to SIRS.

Biliary etiology has proven to be a significant risk for death in our study (*p* = 0.01), we had no alcohol induced SAP compared with other studies which showed a higher rate for alcohol-induced AP compared with biliary-induced AP for our country, but this was a single year study with a low number of patients [18]. One other reason for this was the reluctance of the patients or their relatives (where the patients were intubated before admission to the hospital) to admit alcohol consumption.

Hypertriglyceridemia was not a death risk factor for the patients with SAP, plasmapheresis was performed during their stay in ICU and although these patients had persistent organ failure, 80% survived.

Prediction of mortality in a univariate logistic regression was significant for inflammatory factors NLR and PLR. NLR was tested before and could predict the death of the patients with AP on a cut-off value of 12.2 with an AUROC of 0.851 and a *p*-value lower than 0.001 [9]. In our study, NLR exhibited a cut-off value of 12.4, but a lower AUROC of 0.70, this could be due to the fact that in other studies the population was mixed with severe but also moderate and mild AP. Other findings in a similar setup of mixed severe and mild AP had a cut-off value of NLR = 16.6, AUROC 0.823 [10].

PLR had a cut-off value of 127 in our study, higher than 149 in Zhou et al. study, but with a close AUROC and a statistical significance of 0.75 versus 0.71, *p* = 0.02 versus 0.04 [9].

We found a new model for predicting mortality, composed from NLR above 12.4, PLR above 127, and age above 52 years old, that provided us a good performance, AUROC = 0.95.

C-reactive protein had significantly increased values at hospital admission for the non-survival patients compared with the value for survivals, but CRP was not measured for all the cases at admission, also the follow-up measurements intervals for CRP values were conducted differently for every patient.

Our study limitations are the small cohort and the exclusion of mild and moderate forms, but with a power of 80%, we state that our results are significant and our model for predicting mortality in severe acute pancreatitis is a promising one for clinical practice. The small number of patients with SAP is not abnormal for studies regarding risk factors for morbidity and mortality across literature, Amalio et al. found 108 patients during a twenty years investigation (admitted to the ICU in a referral hospital) [15], Isenman et al. had 158 patients during a 15 years period [5], Park et al. enrolled 52 patients in a 10 years period in a referral hospital [6]. Our study—with 42 patients across 3 years—represents a good cross-sectional perspective.

## 5. Conclusions

Multiple persistent organ failure, age above 50, higher values of CRP, and surgery were risk factors for death in the patients with severe acute pancreatitis admitted to the intensive care unit of our hospital. Prediction of mortality in a univariate logistic regression was significant for inflammatory factors such as the neutrophils-lymphocytes ratio and the platelet-lymphocytes ratio. The model design from combining the neutrophils-lymphocytes ratio, platelets-lymphocytes ratio, and age was the best for predicting mortality in severe acute pancreatitis. Hypertriglyceridemia was not an aggravating risk factor for death.

The early deaths and late deaths of the patients admitted to the intensive care unit with severe acute pancreatitis had different profiles and different mechanisms and should prompt a new classification of severe acute pancreatitis.

Early deaths were more common in our study than late deaths, an indicator of how severe cases were included in our study and a possible explanation for the high death rate.

From our study, we found that “early-death” patients were more likely to die without a known etiology of severe acute pancreatitis mostly because there was no time to find one, had multiple organ failure, and less surgical procedures when compared with the “late-death” patients.

## Figures and Tables

**Table 1:** Patients’ demographics and clinical variables in terms of dead/survival groups.

Variable/Outcome	Survivals (n = 21)	Deaths (n = 32)	*p*-Value
Age, years	47.2 ± 12.4	62.9 ± 12.7	<0.0001
Male n (%)	12 (57.1%)	21 (65.6%)	0.94
Etiology			
Biliary n (%)	13 (61.9%)	18 (56.2%)	0.68
Hypertriglyceridemia n (%)	4 (19.0%)	2 (6.2%)	0.15
Other n (%)	4 (19.0%)	12 (37.5%)	0.30
Organ failure Single/Multiple			
Renal n (%)	3 (14.2%)	3 (9.3%)	0.58
Respiratory n (%)	15 (71.4%)	3 (9.3%)	0.08
Circulatory n (%)	3 (14.2%)	3 (9.3%)	0.58
Multiple n (%)	0	22 (68.75%)	<0.0001
Total hospitalization days	17 (12.2–22.8)	4.5 (3–16.0)	0.01
Days before admission to ICU	2 (1–5)	2 (1–6)	0.58
Days in ICU	7 (1–9.2)	2.5 (1–14)	0.05
Surgery n (%)	5 (23.8%)	20 (62.5%)	0.009
Lipase *	7.2 (4.9–15)	16.1 (5.7–40.2)	0.91
CRP *	6.5 (4.5–8.7)	34.8 (23.8–42.2)	<0.0001
NLR	10.3 (7.5–11.7)	14.2 (11.9–16.7)	0.01
PLR	121 (119.1–336.8)	200.0 (78.8–293.5)	0.20
CTSI	5 (4.0–5.5)	6 (4–7)	0.33
APACHE II (1st day ICU)	6 (3.9–9.0)	11 (6.0–23)	0.004

* Ratio between the measured value and the maximum normal value of the assessing equipment used in the study; ICU—intensive care unit; CRP—C-reactive protein; NLR—neutrophils-lymphocytes ratio; PLR—platelets-lymphocytes ratio; CTSI—computed tomography severity index, APACHE II—Acute Physiology and Chronic Health Evaluation.

**Table 2:** Variables comparison between patients who died after 14 days and before 14 days of hospitalization.

Variable	>14 Days (n = 10)	≤14 Days (n = 22)	*p*-Value
Age (mean)	65.7± 11.5	62 ± 12.5	0.61
Etiology			
Biliary n (%)	8 (80%)	11 (50.0%)	0.07
Hypertriglyceridemia	2 (10.0%)	0	0.51
Other n (%)	2 (10.0%)	11 (50.0%)	0.01
Organ failure			
Single n (%)	5 (50.0%)	5 (22.7%)	0.48
Multiple n (%)	5 (50.0%)	17 (77.3%)	0.04
Days before admission to ICU	22 (14.3–50.2)	1 (1–2)	0.0001
Surgery	10 (100%)	12 (54.5%)	0.0001
Lipase *	13.7 (2.8–62.7)	17.0 (12.7–35.4)	0.80
CRP *	47.7 (30.0–65)	34.8 (22.6–42.0)	0.4
NLR	9.6 (4.4–18.0)	14.5 (4.4–18.0)	0.16
PLR	181 (91.0–199.0)	185.5 (145.0–279.0)	0.62
CTSI	3.5 (4.0–7.0)	6 (2.0–8.0)	0.45

* Ratio between the measured value and the maximum normal value of the assessing equipment used in the study; ICU—intensive care unit; CRP—C-reactive protein; NLR—neutrophils-lymphocytes ratio; PLR—platelets-lymphocytes ratio; CTSI—computed tomography severity index.

**Table 3:** Performance of different variables for predicting death in acute severe pancreatitis.

Variable	Cut-off	AUROC (95%CI)	*p*	Se	Sp	PPV	NPV	LR+	LR−
NLR	12.4	0.70 (0.56–0.82)	0.06	65.6%	80.9%	84.0%	60.1%	3.45	0.42
PLR	127	0.71 (0.57–0.83)	0.04	81.2%	71.4%	81.2%	71.4%	2.84	0.26
Age	52	0.80 (0.56–0.89)	<0.001	81.2%	80.9%	86.7%	73.9%	4.27	0.23

NLR—neutrophils-lymphocytes ratio; PLR—platelets-lymphocytes ratio; *p*—*p*-value, Se—sensibility, Sp—specificity, PPV—positive predictive value, NPV—negative predictive value, LR+—likelihood ratio +, LR−—likelihood ratio −.

## Data Availability

The data presented in this study are available on request from the corresponding author. The data are not publicly available due to hospital policies.
